# Clinical validation of the accuracy of an intra-operative assessment tool using 3D ultrasound compared to histopathology in patients with squamous cell carcinoma of the tongue

**DOI:** 10.1007/s00405-024-08753-3

**Published:** 2024-06-03

**Authors:** N. M. Bekedam, E. L. Koot, E. M. V. de Cuba, M. J. A. van Alphen, R. L. P. van Veen, L. H. E. Karssemakers, L. E. Smeele, M. B. Karakullukcu

**Affiliations:** 1 Department of Head and Neck Surgery and Oncology, Netherlands Cancer Institute, Antoni van Leeuwenhoek, Antoni van Leeuwenhoek, Amsterdam, The Netherlands; 2 Academic Centre of Dentistry Amsterdam, Vrije Universiteit, Gustav Mahlerlaan 3004, Amsterdam, 1081 LA The Netherlands; 3 Department of Pathology, Netherlands Cancer Institute, Antoni van Leeuwenhoek, Amsterdam, The Netherlands; 4 Department of Head and Neck Surgery and Oncology, Cancer Institute, Antoni van Leeuwenhoek, Verwelius 3D Lab, Amsterdam, The Netherlands; 5 Department of Head and Neck Surgery and Oncology, Plesmanlaan 121, 1066 CX Amsterdam, The Netherlands

**Keywords:** 3D Ultrasound, Histopathology, Oral cancer, Resection margin, Squamous cell carcinoma, Tongue

## Abstract

**Background:**

Histopathological analysis often shows close resection margins after surgical removal of tongue squamous cell carcinoma (TSCC). This study aimed to investigate the agreement between intraoperative 3D ultrasound (US) margin assessment and postoperative histopathology of resected TSCC.

**Methods:**

In this study, ten patients were prospectively included. Three fiducial cannulas were inserted into the specimen. To acquire a motorized 3D US volume, the resected specimen was submerged in saline, after which images were acquired while the probe moved over the specimen. The US volumes were annotated twice: (1) automatically and (2) manually, with the automatic segmentation as initialization. After standardized histopathological processing, all hematoxylin-eosin whole slide images (WSI) were included for analysis. Corresponding US images were found based on the known WSI spacing and fiducials. Blinded observers measured the tumor thickness and the margin in the caudal, deep, and cranial directions on every slide. The anterior and posterior margin was measured per specimen.

**Results:**

The mean difference in all measurements between manually segmented US and histopathology was 2.34 (SD: ±3.34) mm, and Spearman’s rank correlation coefficient was 0.733 (*p* < 0.001). The smallest mean difference was in the tumor thickness with 0.80 (SD: ±2.44) mm and a correlation of 0.836 (*p* < 0.001). Limitations were observed in the caudal region, where no correlation was found.

**Conclusion:**

This study shows that 3D US and histopathology have a moderate to strong statistically significant correlation (*r* = 0.733; *p* < 0.001) and a mean difference between the modalities of 2.3 mm (95%CI: -4.2; 8.9). Future research should focus on patient outcomes regarding resection margins.

**Supplementary Information:**

The online version contains supplementary material available at 10.1007/s00405-024-08753-3.

## Introduction

Oncologic surgery of the tongue aims to completely remove the tumor, including a resection margin, while preserving the tongue’s functionality. The margins are often reported as close, meaning less than 5 mm, following the UK Royal College of Pathologists guidelines [1]. Since close margins are associated with a higher chance of recurrence and lower survival rates [2–5], these margins may necessitate adjuvant therapy such as (chemo) radiation or additional surgery.

Histopathological assessment is the gold standard analysis for tongue cancer. Frozen section analysis could be applied, but the sensitivity is insufficient [6]. A drawback of histopathological assessment is the processing time of this assessment. Ultrasound is one of the techniques that can be utilized to assess the resected specimen intra-operatively. Brouwer de Koning et al. demonstrate that ex-vivo ultrasound (US) deep margin assessment exhibits a high correlation with histopathology and has a good agreement [7]. Another study shows that intra-oral US combined with ex-vivo margin assessment by US reduces the number of close margins [8]. A limitation of these studies is the likelihood of a mismatch in measurement location, as the position and orientation of the measured margin cannot be validated. Accurate correlation analysis necessitates precise alignment of the US and histopathology measurements. The 2D US is subject to operator variability as this imaging technique depends on the observer’s experience and the subjectivity of manual distance measurements. In a previous study, we implemented a motorized system to control the movement of the US probe [9]. This motorized 3D US overcomes these problems as no operator is required for acquisition. Furthermore, distances between annotated regions can be automatically computed, reducing the subjectivity of measurements.

The specimen and tumor regions in a 3D US volume can be segmented to obtain 3D models of these targets. The resection margin can then be evaluated in all directions by calculating the distance between the surfaces of the tumor and the specimen. This model enables measuring a distance at a specific location and direction in the 3D US volume. After the acquisition, the corresponding location and direction within the slide can be found by slicing the resected specimen in histopathological slides parallel to the US images. This approach enhances the certainty of validating distance measurements between US and histopathology, particularly regarding the location.

This study aims to assess the agreement between the distance measurements by 3D US and histopathology. In ten patients diagnosed with and treated for tongue squamous cell carcinoma (TSCC) were multiple distance measurements performed in the US and histopathology images. The measurements were evaluated by computing statistical correlations and Bland Altman plots.

## Method

### Study population and materials

This study prospectively included ten patients diagnosed with TSCC and treated with curative surgery. A mucosal margin of 10 mm surrounding the tumor was annotated by the surgeon prior to incision. Intraoral in-vivo imaging of the tumor was performed during the excision with a 18 MHz transducer (X18L5s, BK Medical, Denmark). Additional tissue was resected immediately in case in-vivo imaging showed close margins. Sutures marked the resected specimen for anatomical orientation during this surgery. Additionally, up to three intravenous (IV) cannulas of 16 gauge were inserted through the resected specimen in the longest dimension of the specimen as fiducials. 3D US acquisition was performed using a 10 MHz transducer (I14C5T, BK Medical, Denmark) on a BK5000 US system (software version: 5.148.18234.26, license: OEM interface, BK Medical, Denmark). 3D acquisition was enabled by applying the transducer to a motorized system (Master 2s, NEJE, China) [9]. Histopathology data consisted of both macroscopic and microscopic images. The macroscopic light images were taken from the specimen throughout the process, from fixating to slicing. A digital whole slide image (WSI) was obtained from each histopathological slide using a digital scanner (software version 3.2.1, Pannoramic 1000, 3DHistech, Budapest, Hungary). The WSIs were exported using SlideViewer (version 2.6, 3DHistech Ltd., Budapest, Hungary). The image analysis for both 3D US and histopathology was performed using 3DSlicer (version 5.2.1), a free, open-source software application for medical image computing [10]. Statistical analysis was performed in SPSS (version 29.0, IBM, Chicago, IL, USA) [11]. This study was approved by the medical ethics committee (NL-78804.031.21). All included patients signed an informed consent before enrollment.

### Data acquisition

**Fig. 1:**
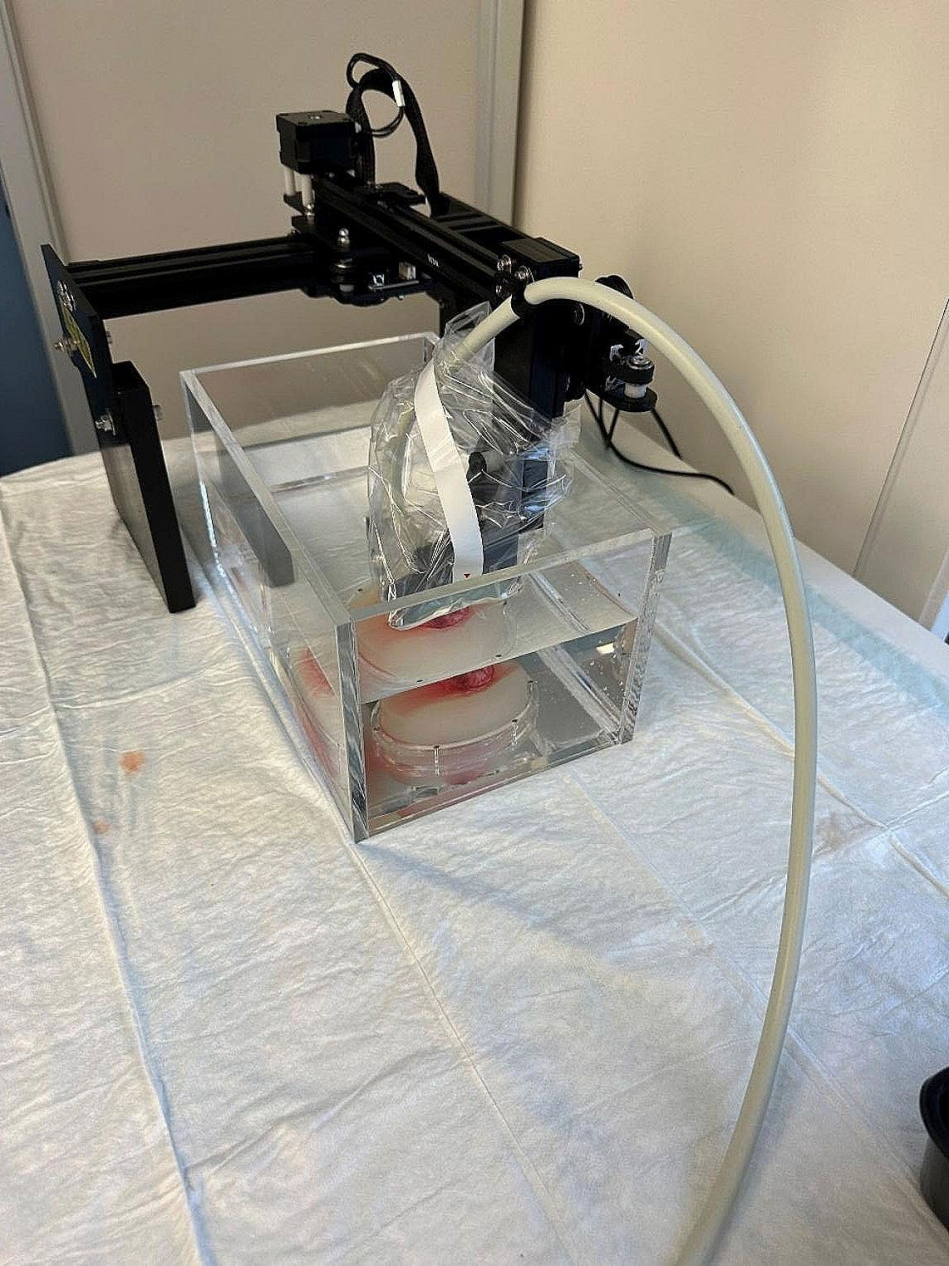
The motorized 3D ultrasound setup. The black system is the motorized rails. The transducer is placed below the saline surface so that the saline can transmit the sound waves [9]

Motorized 3D US acquisition was performed following the method by Bekedam et al. [9] as described above, of which the setup is shown in Fig. 1. Summarized, the resected specimen, including the IV cannulas, was placed on a US gel pad in a saline-filled container. The IV cannulas function as fiducial markers in the US and histopathological images. The US transducer was positioned below the saline surface so that the saline can transmit the sound waves. The motorized system continuously moved the transducer along the specimen while the US system acquired all the images. The images were reconstructed into a 3D US volume with a slice spacing of 0.5 mm based on the frame per second of the US acquisition and the motor speed.

After the intra-operative US acquisition, the specimen with the IV cannulas inserted was brought to the Department of Pathology. Following standard protocol, the specimen was fixated and inked for specimen orientation. Post-inking, the inserted IV cannulas were removed, leaving an artificial hole in the specimen. Next, the specimen was equally cut into four mm thick slices along the longest dimension of the specimen. TruSlice (Cellpath, Newtown, United Kingdom), a cut-up system to limit dissection inaccuracies was utilized. If a slice did not fit the cassette, the slice was cut into several parts. The processing continued with a standard protocol of paraffin embedding. From the four mm paraffin slices, a four µm slide was cut and mounted on glass. All slides were stained with hematoxylin and eosin (H&E) for conventional pathological assessment. This assessment was performed on digitized H&E slides.

### Tumor annotation

The specimen and tumor in the 3D US volume were annotated twice: (1) automatic segmentation by a deep learning network [12] and (2) manual segmentation by a technical physician with the automatic segmentation as initialization. This initialization minimizes the annotation variability [13]. A head and neck pathologist manually annotated the WSIs. Both annotators were unaware of the other modality to avoid potential bias.

### Measurements

For a correct comparison of measurements in 3D US and histopathology, it was necessary to find the US images corresponding to the obtained WSI. The corresponding US image was found in two steps. First, the length of the annotated specimen in the 3D US volume was measured and divided by the number of four mm thick histopathological slices. Second, based on the fiducial markers, the linearity of distances between the fiducial markers provided more certainty about the found US image corresponding to the WSI. Finally, the best corresponding US image to match the WSI was found with a visual inspection of histopathological features and the fiducial markers.

**Fig. 2:**
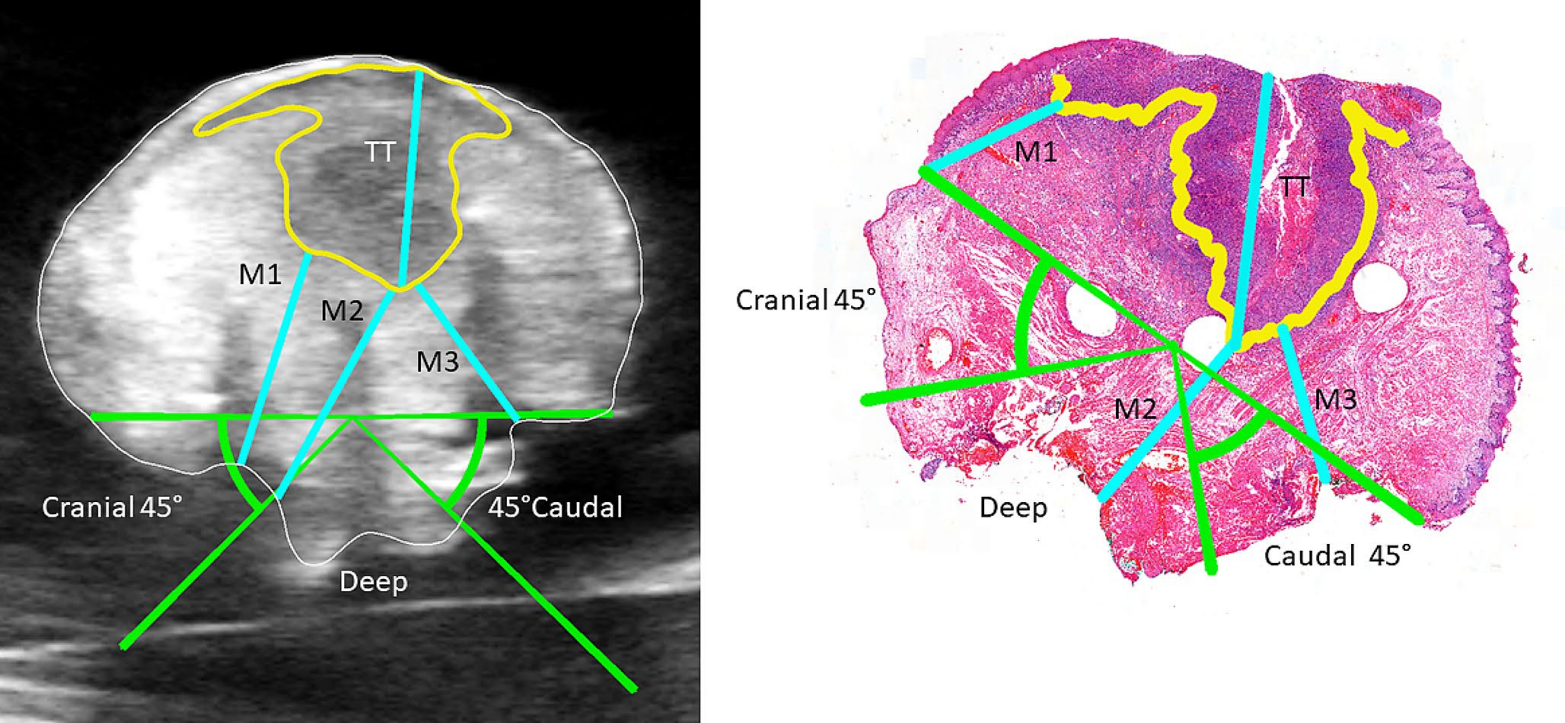
Left the ultrasound image corresponding to the Whole Slide Image from histopathology on the right. The tumor is annotated in yellow, the cranial, deep, and caudal regions are green, and the distance measurements are blue. The three holes in the whole slide image result from the inserted IV cannulas. These inserted IV cannulas created a hypoechoic shadow in the ultrasound image. The hypoechoic characteristic of tumor tissue on ultrasound imaging was also caused by the tumor-associated inflammation and stromal reaction, not only the tumor cells. TT = Tumor Thickness; M1 = Cranial margin; M2 = Deep margin; M3 = Caudal margin

Then, the resection surface in both US and WSI images was identified to be measured more objectively using the following method by both observers. A line was drawn between the mucosal edges in both the WSI and the corresponding US image. The mucosal edges were visible in the WSI. For US, the 3D shape of the annotated specimen and the sutures helped to distinguish the transition from the mucosal surface to the resection surface. From the middle of this line, a new line at a 45-degree angle was created twice to divide the resection surface into three regions. Most specimens were cut orthogonal to the anterior-posterior dimension, so the three regions were caudal, deep, and cranial. Figure 2 shows an example of the WSI and the corresponding US image, including the line between mucosal edges and the three regions.

Four measurements were conducted for each image containing tumor: the closest distance from the tumor annotation to the resection surface (the resection margin) in all three regions and the tumor thickness (TT). The TT is the maximum thickness of the tumor. Depth of invasion (DOI) is pathologically measured from the basement membrane of the nearest normal mucosa. From here, the deepest invasion of tumors is the DOI.The basement membrane is not visible in radiological modalities such as US (4). Therefore, DOI was not included in the measurements. The WSIs from the histopathological slices, which did not fit in the cassette during paraffin processing, were digitally resembled in the original slice. Besides the four measurements in each image, the remaining resection margins in the anterior and posterior regions were also measured. For histopathology, the number of tumor-free slices to the anterior or posterior cap was multiplied by the slice thickness of four mm. For US, the closest distance from the tumor annotation to the anterior or posterior end of the specimen annotation was measured. Again, both observers were blinded to the other modality.

### Statistical analysis

Data was tested for normality. The correlation between the measurements by 3D US and histopathology was evaluated using Pearson’s correlation coefficient in the case of normal distribution. Otherwise, Spearman’s rank correlation coefficient was used. Correlation between modalities was considered significant if *p* < 0.05. The agreement between the modalities was estimated with a Bland Altman plot, which presents the difference between the modalities against the mean of the modalities [14]. The correlation and the agreement were computed for all measurements and per region: thickness, caudal, deep, cranial, anterior, and posterior.

## Results

A total of 10 patients were included in this study. One patient was excluded and replaced with another because histopathological assessment did not show any tumor in the resected specimen. The mean age of the included patients was 66 years (min: 36 – max: 86 years), and 7 out of 10 were male. The tumors were located on the left side (*n* = 5) and right side (*n* = 5) of the tongue border and clinically staged as T1 (*n* = 5), T2 (*n* = 3), and T3 (*n* = 2). Intraoral in-vivo imaging did not show close margins during the excision, so none of the patients received an immediate re-resection. Histopathological analysis showed a close margin in 7 out of 10 patients. From the ten resected specimens, 53 histopathological slices contained tumors. In some slices, the three inserted IV cannulas were not visible due to the shape of the resected specimen.

The data was not normally distributed. The Spearman’s rank correlation coefficient between the measurements by 3D US and histopathology is shown in Table 1. For all measurements combined, the manual segmentation of 3D US volumes resulted in a higher correlation with histopathology than the automatic segmentation. Specifying per region, the best correlation based on manual segmentation was found in the tumor thickness (*r* = 0.836; *p* < 0.001). This region achieved the best correlation based on automatic segmentation as well (*r* = 0.865; *p* < 0.001).

**Table 1:** The Spearman’s rank correlation coefficient between the measurement by 3D ultrasound, for both automatic and manual segmentation, and histopathology

	Spearman correlation coefficient between 3D Ultrasound and histopathology	
Region	Automatic segmentation (*p*-value)	Manual segmentation (*p*-value)
Tumor thickness	**0.865** (***p* < 0.001**)	**0.836** (***p* < 0.001**)
Caudal	0.240 (*p* = 0.165)	**0.472** (***p* = 0.007**)
Deep	**0.785** (***p* < 0.001**)	**0.831** (***p* < 0.001**)
Cranial	**0.693** (***p* < 0.001**)	**0.721** (***p* < 0.001**)
Anterior	**0.669** (***p* = 0.035**)	0.357 (*p* = 0.281)
Posterior	0.238 (*p* = 0.508)	**0.764** (***p* = 0.006**)
All measurements	**0.657** (***p* < 0.001**)	**0.733** (*p*** < 0.001**)

The measurements were both divided per region and all combined. The correlation is considered significant if *p* < 0.05, indicated in bold

**Fig. 3:**
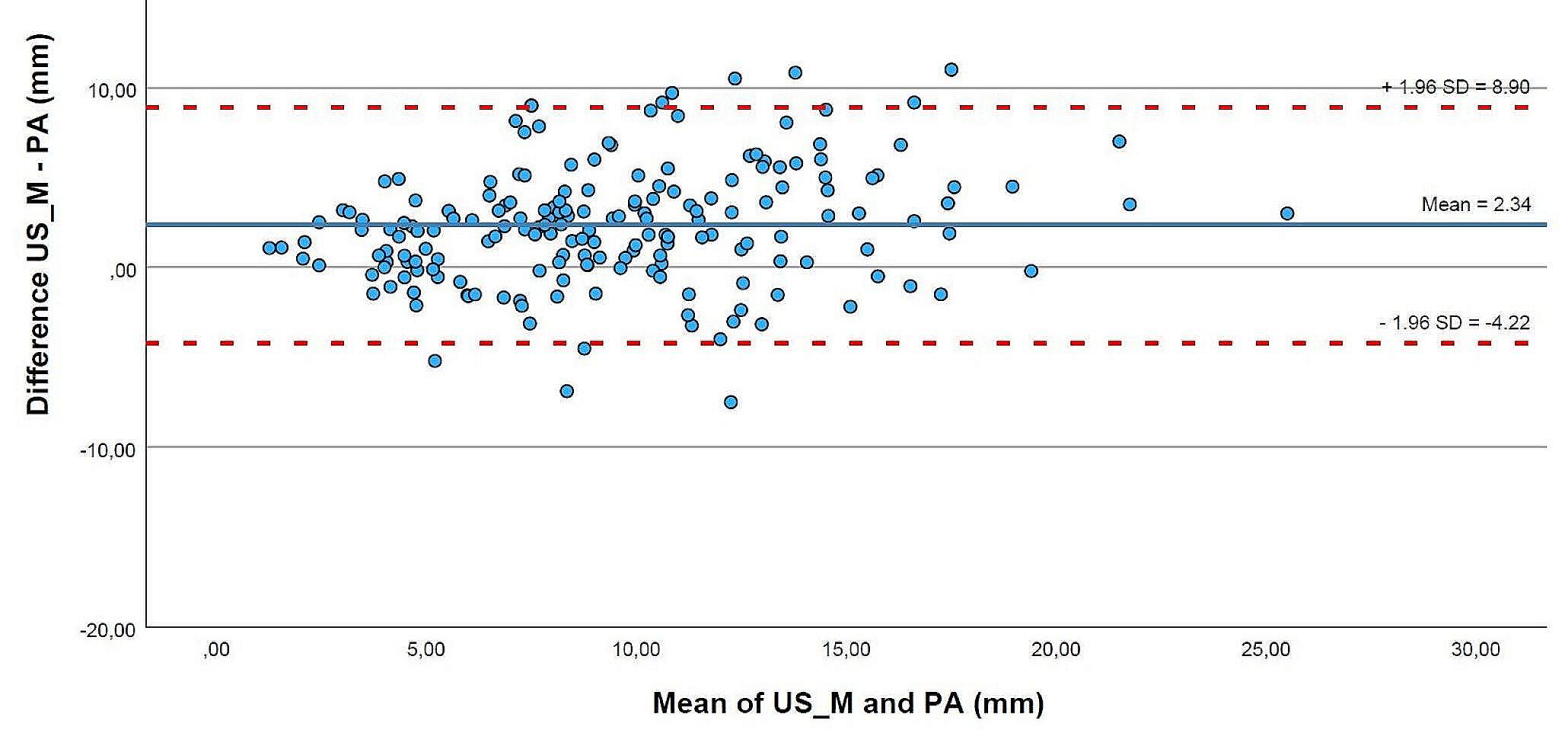
Bland Altman plot of the difference between all measurements (*n* = 188) by ultrasound and histopathology against the mean of both measurements. Ultrasound measurements are based on the manual segmentation of the volume. The blue horizontal line is the mean difference between ultrasound and histopathology. The two red dashed lines are the lower and upper limits of agreement of the 95% confidence interval. US_M = manual segmentation of ultrasound, PA = histopathology, SD = standard deviation, mm = millimeter

**Fig. 4:**
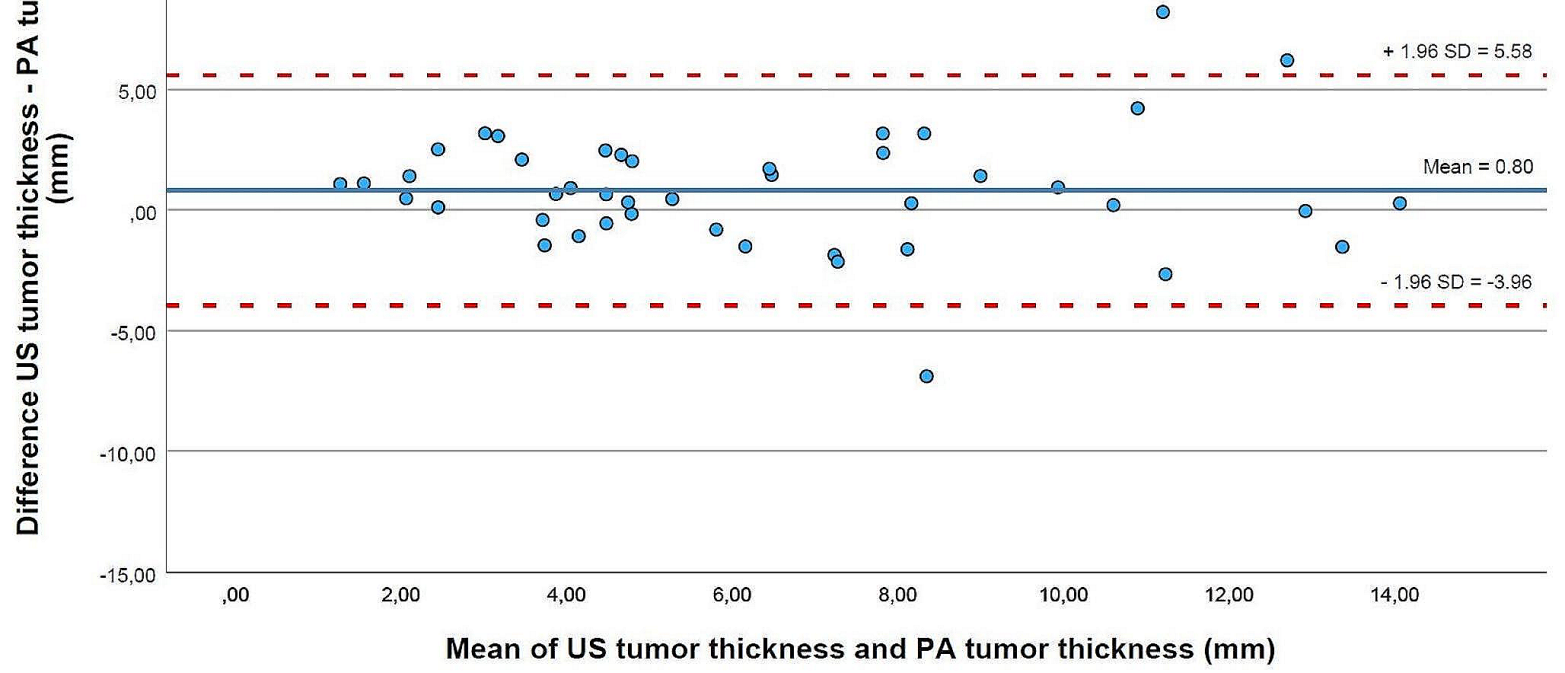
Bland Altman plot of the difference between the measurements of tumor thickness (*n* = 44) by ultrasound and histopathology against the mean of both measurements. Ultrasound measurements are based on the manual segmentation of the volume. The blue horizontal line is the mean difference between ultrasound and histopathology for tumor thickness. The two red dashed lines are the lower and upper limits of agreement of the 95% confidence interval. US = ultrasound, PA = histopathology, SD = standard deviation, mm = millimeter

The mean difference between the manual segmentation of 3D US and histopathology in all distance measurements was 2.34 mm (95% Confidence Interval (CI): -4.22; 8.90), as shown in Fig. 3. The agreement between the two modalities of the TT, which was the smallest difference found with 0.81 mm (95%CI: -3.96; 5.58), was shown in Fig. 4. For automatic segmentation, the mean difference in all measurements was 2.98 mm (95%CI: -4.78; 10.75), with the smallest difference between the modalities found in the TT as well, 0.73 mm (95%CI: -4.39; 5.85). Table 2 shows the 95%CI for manual and automatic segmentation for the other regions.

**Table 2:** The agreement per region between the measurements by 3D ultrasound, for both automatic and manual segmentation, and histopathology

	Agreement between 3D ultrasound and histopathology	
Region	Mean difference with automatic segmentation in mm (95%CI)	Mean difference with manual segmentation in mm (95%CI)
Tumor thickness	0.73 (-4.39; 5.85)	0.81 (-3.96; 5.58)
Caudal	4.69 (-5.11; 14.49)	3.11 (-4.87; 11.1)
Deep	3.07 (-0.90; 7.03)	2.62 (-1.67; 6,91)
Cranial	3.41 (-2.94; 9.77)	2.73 (-2.87; 8.34)
Anterior	4.21 (-3.34; 11.76)	1.87 (-8.34; 12.08)
Posterior	2.55 (-12.48; 17.58)	3.46 (-5.23; 12.14)

Detailed analysis showed significant differences in measurements of patient eight between the 3D US and histopathology. Upon further investigation, the surgeon stretched out and pinned patient 8’s specimen prior to delivery at the Department of Pathology for fixation. Stretching out the specimen resulted in outliers in this particular case. After analysis excluding the measurements from patient eight, improved results were found, as shown in Appendix A. The correlation between manual segmentation of 3D US and histopathology improved to 0.756 (*p* < 0.001) and for automatic segmentation to 0.717 (*p* < 0.001). The agreement between the two modalities, excluding the measurements of patient eight, resulted in 2.27 mm (95%CI: -3.88; 8.44) for manual segmentation and 2.93 mm (95%CI: -4.15; 10.02) for automatic segmentation.

It is worth highlighting that the hypoechoic characteristic of tumor tissue on US imaging was also caused by the tumor-associated inflammation and stromal reaction, not only the tumor cells. This is visible in Fig. 2; the tumor annotation was less wide than the dark purple tumor process in the WSI. The tumor annotation in the US image contained a hypoechoic wider area matching this tumor-associated inflammation and the stromal reaction of the WSI.

## Discussion

In this study, we investigated the agreement between the distance measurements by 3D US and histopathology. The tumor region in 3D US was segmented manually and automatically. The main finding of this study showed that manual segmentation of 3D US resulted in the best agreement with histopathology of 2.34 mm (95%CI: -4.22; 8.90) and a corresponding correlation of 0.729 (*p* < 0.001). For automatic segmentation, the agreement between 3D US and histopathology was 2.98 mm (95%CI: -4.78; 10.75) with a correlation of 0.648 (*p* < 0.001). These results suggested that obtaining a > 5 mm histopathological margin requires an additional 2.34 mm when measured ex-vivo by 3D US on average for all distance measurements.

Novel aspects of our study were the 3D US technique and the comparison of multiple measurements within one slice. Comparison with previous studies in the literature required specification of the regions; to our knowledge, we were the first to use 3D US for resected tongue specimen assessments. Our results showed a considerable higher mean difference between US and histopathology than the 0.05 mm with a correlation of 0.79 (*p* < 0.01) described by Brouwer de Koning et al. [7]. They only measured the deep margin. Specifying our results for the deep margin, we obtained an agreement of 2.61 mm (95%CI: -1.68; 6.90) with a correlation of 0.826 (*p* < 0.0001) with histopathology. This result confirmed the positive correlation between US and histopathology. Several studies investigated the TT or depth of invasion as well. One study reports a significant correlation of 0.67 (*p* < 0.001) between TT measured by US and histopathology [15]. Caprioli et al. show a significant relationship between US and histopathology for the depth of invasion of 0.84 (*p* < 0.0001) [16]. Additionally, their agreement between the two modalities in measuring the depth of invasion was 0.7 mm (95%CI 0.26; 1.16), similar to our agreement for TT. Our results showed correlations and agreements between histopathology and 3D US comparable with studies that investigated 2D US.

The strength of this study was the objective method of measuring the TT and the three margins per slice. The 3D visualization of the resected specimen by US enabled slice matching with histopathology. Based on the number of WSI and the added fiducial markers, the US image corresponding to the WSI could be found more securely. Also, the margins were measured more objectively as the resection surface was divided into three regions following the same method for US and histopathology. This method is not used in clinical routine, but for this study, it is more objective and helps to minimize the variability in measurements. Another strong point of the study was blinding the two observers who analyzed the US and WSI separately.

The limitations of this study were mainly related to the differences in measuring between the two modalities. First of all, the measurements by US were performed during surgery on freshly resected tissue, while histopathology was measured after fixation and slicing. Besides the time difference, fixating and paraffin embedding were known causes for tissue to shirk from 11.3% up to 35% [17–20]. These causes are embedded in the routine clinical practice. Second, cutting the specimen into four mm and four µm slices resulted in tissue deformation. Another limitation was that the cut tissue did not fit into the cassette for paraffin embedding. Although digital resembling the half WSIs maintained the inclusion of these data, perfect fitting of the two halves cannot be guaranteed and might influence the measurements slightly. Lastly, the mucosal-resection surface transition is hard to distinguish by US. For example in Fig. 2, where this transition was indicated elsewhere resulting in different region compared to histopathology. We could not prevent the first limitations as standardized histopathological protocols had to be followed.

Observer variability among pathologists is a well-known phenomenon [21–23]. In our study, all histopathological annotations were performed by a single pathologist to eliminate interobserver variability. Besides histopathology, the 3D US annotations in this study were subject to observer variability. Moreover, annotations included both tumor and specimen regions. As measurements were performed from one annotation to the other, the variability was a source of error that could influence these measurements on both sides. Therefore, the proposition by Tizhoosh et al. to apply different AI models to tackle this variability problem could be a solution to minimize this problem in the future for histopathology and US [22].

Results showed that the correlations between the measurements by 3D US and histopathology were lower in the anterior and posterior regions compared to the other regions. These lower correlations were probably due to the limited observations in the anterior and posterior regions. Except for one specimen, all specimens had the longest dimension in the anterior-posterior direction, meaning histopathological slices were cut perpendicularly to this direction. This method means the anterior and posterior margins were measured per specimen instead of per slice, resulting in fewer measurements. Additionally, these margins were measured by counting the tumor-free slices, which had a four mm slice thickness, to the end caps of the specimen. This rough step size was more sensitive to mismatch the 0.5 mm slice thickness of the 3D US.

The lower correlation in the caudal tumor region could be explained by heterogeneous tissue such as the sublingual glands and submandibular glands, arteries, nerves, and connective tissue [24]. These different structures made it more challenging to distinguish the tumor from healthy tissue in US images, whereas, in the other regions, the surrounding healthy tissue mainly consisted of tongue muscle. This insecurity may suggest that 3D US is not feasible in margin assessment in the vicinity of the floor of the mouth.

Interpreting the Bland Altman plots indicated that an average distance of 2.3 mm extra was measured with 3D US compared to histopathology. To obtain the > 5 mm histopathological margin, intraoperative 3D US measurements should be 7.3 mm on average. But a histopathological clear margin is based on the final closest margin, which includes the worst measurements, the outliers. The upper limit of the 95%CI of 8.90 mm indicates a false negative margin. Then, no re-resection will be performed resulting in a histopathological margin of < 5 mm. The lower limit of the 95%CI of -4.22 mm indicates that 3D US tends to find a false close margin, by which a re-resection will lead to complete removal of the tumor but more functional impairment. Complete removal with acceptance of more functional impairment is similar to the strategy of compartmental surgery. Missale et al. find that the type of surgery, compartmental or wide local excision, resulted in similar oncological outcomes [25]. They did not include the post-operative quality of life in the analysis, which we assume will be lower for the compartmental surgery. Depending on the surgeon, one could prefer complete tumor removal with more functional impairment or minimizing tissue loss with a chance of a close margin. Patient 8 resulted in outliers after being fixated on a stretched-out matter, negatively influencing the correlation and the agreement between 3D US and histopathology. Excluding the measurements from patient 8 showed a slight improvement in Spearman’s correlation coefficient among the different regions between the modalities. As this specimen was logistically treated differently, we believed the results without this specimen were clinically more reliable.

As in general it is preferred to obtain a larger resection margin rather than an insufficient one, for which we will detail the negative differences between US and histopathology for further understanding. A larger margin measured by histopathology than US could be explained by several reasons. The hypoechoic region, considered as tumor, could be delineated vaguely and challenging the tumor annotation in US more. Examples of those challenges were the tumor-associated inflammation and the glandular tissue in the caudal region, as described above. Secondly, tissue deformation between the US and histopathology measurements occurred. Freshly resected specimens could be more flat during US acquisition than fixated and cut tissue for paraffin embedding in a histopathological cassette. Thirdly, as explained above as limitation, the mucosal-resection surface transition was hard to distinguish by US. In Fig. 2, the cranial margin was larger in US than histopathology due to this transition, but this could occur in the inverse direction as well.

It is noteworthy that there is an ongoing discussion regarding the optimal margin distance for oral tumors. The guidelines from the Royal College of Pathologists were reformulated in 2023 [1]. Suggested margin distances ranged from 2.2 mm to 7.5 mm as oncologically safe [2, 26–28]. The College of American Pathologists states that cut-offs between 3 and 7 mm have been used with success [29].

Our study contributed to the improvement of intra-operative assessment of margins in resected TSCC. A conventional US system was applied in a new setup, and this study clinically validated the agreement between 3D US and histopathology objectively. In this study, we observed that in four measurements of superficial and filamentous tumors, they may not be detected by US due to limited visibility. Conversely, we note a learning curve in identifying glandular tissue and the mucosal-resection surface transition. With these findings as a foundation, broader cohort studies can be undertaken to explore the influence of intraoperative assessment on patient outcomes regarding the margin and, subsequently, its potential to reduce the need for adjuvant therapy.

In conclusion, this study shows that 3D US and histopathology have a moderate to strong statistically significant correlation of *r* = 0.733 (*p* < 0.001) and a mean difference between the two modalities of 2.3 mm (95%CI: -4.2; 8.9). During ex-vivo margin assessment, an additional 2.3 mm on the 3D US should be considered to achieve an average histopathological clear margin of > 5 mm. Future research should focus on patient outcomes regarding resection margins as the dependent variable.

## Electronic supplementary material

Below is the link to the electronic supplementary material.

Supplementary Material 1
