# Supplementary material for: Clinical validation of the accuracy of an intra-operative assessment tool using 3D ultrasound compared to histopathology in patients with squamous cell carcinoma of the tongue
Source: Eur Arch Otorhinolaryngol. 2024 Jun 3;281(10):5455–63. doi: 10.1007/s00405-024-08753-3 (PMC11416359; doi:10.1007/s00405-024-08753-3)
Supplement: Supplementary file 1 — Supplementary Material 1 [file 405_2024_8753_MOESM1_ESM.docx]

Table 3: The Spearman’s rank correlation coefficient between the measurement without the outliers of patient eight by 3D ultrasound for both automatic and manual segmentation and histopathology. The measurements were both divided per region and all combined. The correlation is considered significant if p<0,05, indicated in bold. SD = standard deviation. mm = millimeter.

|  | **Spearman’s rank correlation coefficient between 3D Ultrasound and histopathology excluding patient 8** | |
| --- | --- | --- |
| **Region** | **Automatic segmentation (p-value)** | **Manual segmentation (p-value)** |
| Tumor thickness | **0.843 (p < 0.001)** | **0.878 (p < 0.001)** |
| Caudal | 0.184 (p = 0.341) | **0.352 (p = 0.048)** |
| Deep | **0.786 (p < 0.001)** | **0.784 (p < 0.001)** |
| Cranial | **0.715 (p < 0.001)** | **0.790 (p < 0.001)** |
| Anterior | 0.586 (p = 0.097) | 0.567 (p = 0.087) |
| Posterior | **0.704 (p = 0.034)** | **0.715 (p = 0.020)** |
| All measurements | **0.717 (p < 0.001)** | **0.756 (p < 0.001)** |


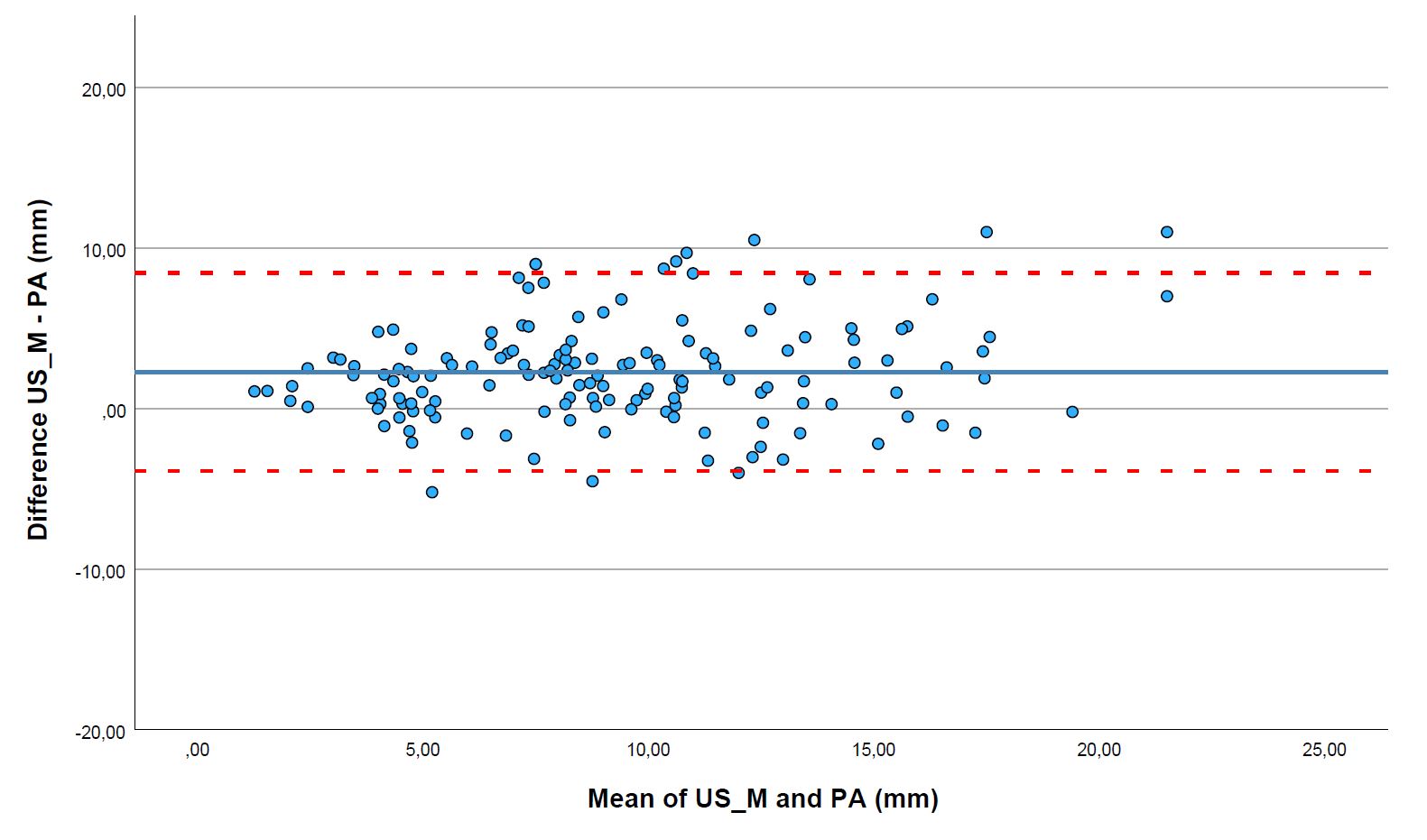


Figure 5: Bland Altman plot of the difference between all measurements (n=149), excluding patient 8, by ultrasound and histopathology against the mean of both measurements. Ultrasound measurements are based on the manual segmentation of the volume. The blue horizontal line is the mean difference (2,28 mm) between ultrasound and histopathology. The two red dashed lines are the lower (-3,88 mm) and upper (8,44 mm) limits of agreement of the 95% confidence interval. US_M = manual segmentation of ultrasound; PA = histopathology.


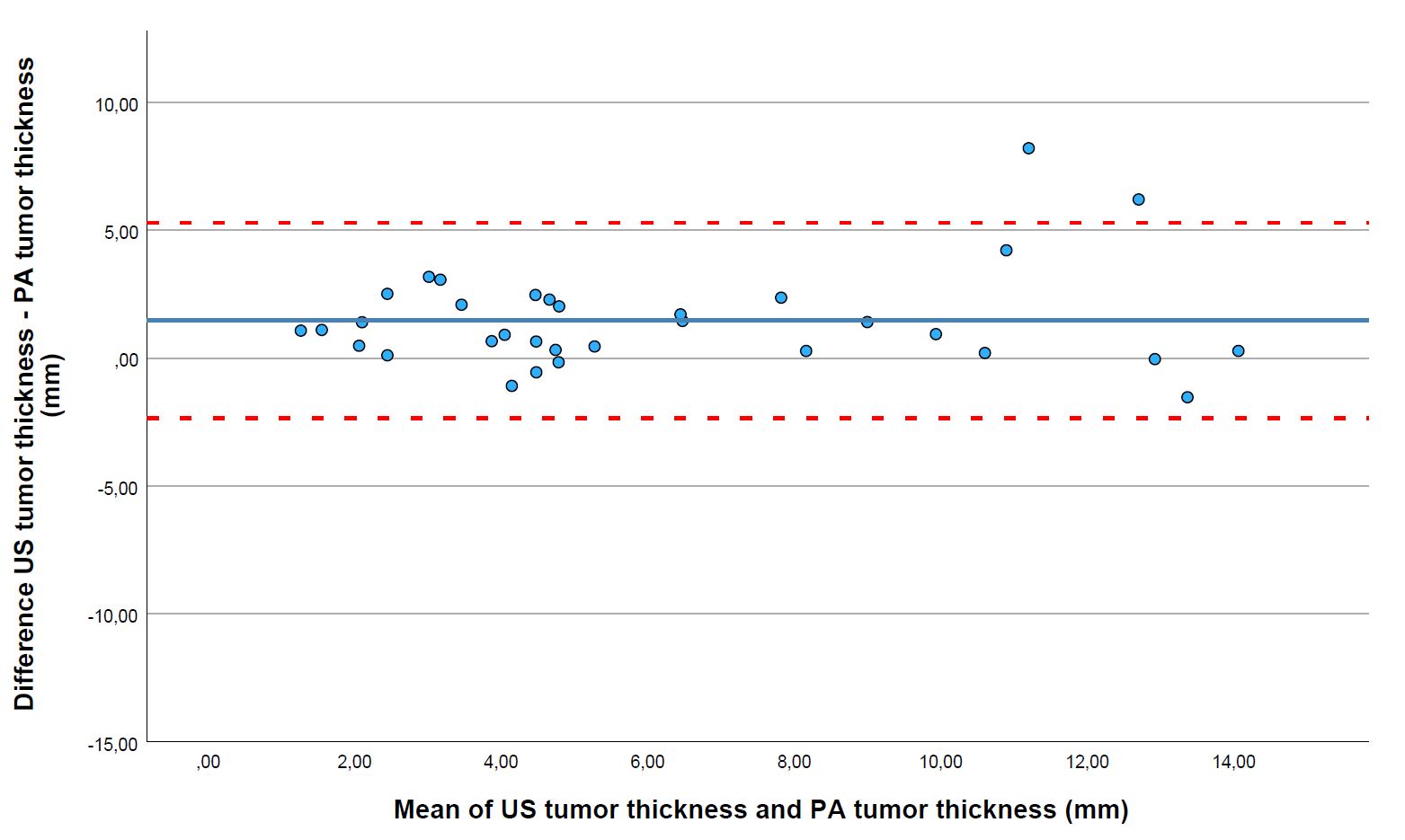


Figure 6: Bland Altman plot of the difference between all measurements (n=36), excluding patient 8, by ultrasound and histopathology against the mean of both measurements. Ultrasound measurements are based on the manual segmentation of the volume. The blue horizontal line is the mean difference (1,47 mm) between ultrasound and histopathology. The two red dashed lines are the lower (-2,34 mm) and upper (5,29 mm) limits of agreement of the 95% confidence interval. US_M = manual segmentation of ultrasound; PA = histopathology.

Table 4: The agreement per region between the measurements, excluding patient 8, by 3D ultrasound, for both automatic and manual segmentation, and histopathology.

|  | **Agreement between 3D ultrasound and histopathology** | |
| --- | --- | --- |
| **Region** | Mean difference with automatic segmentation (95%CI) | Mean difference with manual segmentation (95%CI) |
| Tumor thickness | 1.26 (-3.52; 6.04) | 1.47 (-2.34; 5.29) |
| Caudal | 4.06 (-5.76; 13.89) | 2.85 (-5.10; 10.80) |
| Deep | 2.63 (-1.05; 6.31) | 2.10 (-1.32; 5.53) |
| Cranial | 2.87 (-3.80; 9.54) | 2.08 (-3.43; 7.59) |
| Anterior | 4.51 (-3.25; 12.27) | 2.81 (-5.83; 11.45) |
| Posterior | 4.56 (-4.40; 13.52) | 3.45 (-5.71; 12.61) |
